# Who cares? Implications of care-giving and -receiving by HIV-infected or -affected older people on functional disability and emotional wellbeing

**DOI:** 10.1017/S0144686X13000615

**Published:** 2013-09-04

**Authors:** M. NYIRENDA, M. EVANDROU, P. MUTEVEDZI, V. HOSEGOOD, J. FALKINGHAM, M.-L. NEWELL

**Affiliations:** * Africa Centre for Health and Population Studies, University of KwaZulu-Natal, Somkhele, South Africa.; † Faculty of Social and Human Sciences, University of Southampton, UK.; ‡ Division of Population Health, University College London, UK.; § Faculty of Medicine and Faculty of Social and Human Sciences, University of Southampton, UK.

**Keywords:** South Africa, older people, care-giving, HIV-infected, self-reported health, functional disability, emotional wellbeing

## Abstract

This paper examines how care-giving to adults and/or children and care-receiving is associated with the health and wellbeing of older people aged 50+ in rural South Africa. Data used are from a cross-sectional survey adapted from World Health Organization's Study on Global Ageing and Adult Health (SAGE) conducted in 2009/10 in rural South Africa. Bivariate statistics and multivariate logistical regression were used to assess the relationship between care-giving and/or care-receiving with functional disability, quality of life or emotional wellbeing, and self-rated health status, adjusted for socio-demographic factors. Sixty-three per cent of 422 older people were care-givers to at least one young adult or child; 27 per cent of older people were care-givers due to HIV-related reasons in young adults; 84 per cent of participants were care-recipients mainly from adult children, grandchildren and spouse. In logistic regressions adjusting for sex, age, marital status, education, receipt of grants, household headship, household wealth and HIV status, care-giving was statistically significantly associated with good functional ability as measured by ability to perform activities of daily living. This relationship was stronger for older people providing care-giving to adults than to children. In contrast, care-givers were less likely to report good emotional wellbeing; again the relationship was stronger for care-givers to adults than children. Simultaneous care-giving and -receiving was likewise associated with good functional ability, but about a 47 per cent lower chance of good emotional wellbeing. Participants who were HIV-infected were more likely to be in better health but less likely to be receiving care than those who were HIV-affected. Our findings suggest a strong relationship between care-giving and poor emotional wellbeing via an economic or psychological stressor pathway. Interventions that improve older people's socio-economic circumstances and reduce financial hardship as well as those that provide social support would go some way towards mitigating this relationship.

## Background

Older people in rural South African communities are a vital source of financial, physical and emotional support to children and adults alike (Ardington *et al.* 2010; Hosegood and Timæus 2006; Nyirenda and Newell 2010; Schatz 2007). Due to high adult unemployment levels (Bor *et al.* 2012; Curtis, Bradshaw and Nojilana 2002), co-residence with older people is important for care and living arrangements (Hosegood, Benzler and Solarsh 2006). Most households in rural South Africa are thus multi-generational, defined as households consisting of grandparents, adult children and grandchildren living in the same homestead (Hosegood and Timæus 2005). Older people 50 years and older are usually the main care-givers in such households, with the heavier burden of care usually borne by older women (Burns, Keswell and Leibbrandt 2005; Schatz and Ogunmefun 2007; Ssengonzi 2007).

In developing countries, particularly in Africa, where institutional care facilities are lacking, there is a general expectation that care and support should be flowing from adults and grandchildren to older persons (Aboderin 2005; Sokolovsky 2001). As observed by Aboderin (2004), a combination of declining capacity by young people to provide for older people and society norms changing to emphasising self-reliance in old age, has exposed an increasing proportion of older people in many African countries to destitution and poverty. This apparent decline in the flow of support from younger people may be cause for distress among older people. Despite these changes in intergenerational support relations (Lowenstein 2005), the family remains an important structure in the care by and towards older people. These support relations may vary from positive to negative or be ambivalent (Antonucci *et al.* 2011; Funk 2012), depending on, for instance, age, gender and socio-economic status of the care-giver (Schröder-Butterfill and Fithry 2012). According to the role-enhancement and role-strain perspective, performing this care-giver role could either enhance or weaken the health and wellbeing of older people (Goode 1960).

In a population severely affected by HIV, older people are important providers of long-term personal and health care to their HIV-infected adult offspring and orphaned children (Dayton and Ainsworth 2004; Hill, Hosegood and Newell 2008; Ssengonzi 2007). Hosegood and Timæus (2005) showed that a decade ago about 12 per cent of all households in rural South Africa with at least one older person 60 years and older had experienced the death of an adult child due to AIDS. These adult deaths resulted in ‘skip-generation’ households and contributed to major living arrangement changes, thrusting older people into a care-giver role. Furthermore, older people are not immune to HIV acquisition (Cooperman, Arnsten and Klein 2007; Dougan *et al.* 2004), and an increasing number are ageing with HIV as more adults on HIV treatment survive into older age (Nguyen and Holodniy 2008). A nationally representative survey showed that about 10 per cent of older people aged 50+ in South Africa are HIV-infected (Shisana *et al.* 2009). In our study community the estimated HIV prevalence rate among older people in 2008 was 9.5 per cent, and the HIV incidence rate was 0.5 per 100 person-years of observation (Wallrauch, Bärnighausen and Newell 2010). Older people are thus both sources of care and support and in need of care and support.

In international literature, the provision and receipt of care has been shown to have profound social, physical, financial and emotional impact on the provider and the receiver (Abad-Corpa *et al.* 2012). In a study among Australian adults aged 16–85 years who were care-providers to a relative with mental disorders (Pirkis *et al.* 2010), women and those relatively older were found more likely to be care-givers. Pirkis *et al.* (2010) further found that this care-giving role was associated with a high financial cost and, more crucially, that care-givers' own mental health and wellbeing was severely affected. In a nationally representative sample of older adults aged 50+, those providing a spouse with long-term care, defined as assistance with basic or instrumental activities of daily living of 14 hours or more per week, had a two-fold significantly increased risk of developing cardiovascular disease (Capistrant *et al.* 2012). On the other hand, a positive impact of care-giving on the quality of life of older care-givers has been reported that negates the care-giving burden (Ratcliffe *et al.* 2013). Reliable information on the effect of care-giving on the health and wellbeing of older people in sub-Saharan Africa is, however, lacking.

Information from pioneer cash transfer programmes in some developing countries, including Brazil and South Africa, indicate that the old-age pension system has been successful in reducing income poverty of households with pensioners relative to non-pensioner households (Lloyd-Sherlock, Saboia and Ramírez-Rodríguez 2012; Lloyd-Sherlock *et al.* 2012*a*). What remains unclear is whether giving these old-age pensions to older people enhances their health status (Lloyd-Sherlock *et al.* 2012*b*). Having an older person in the household with a cash income source, such as the old-age pension grant, is important since it may facilitate the migration of adults in search of employment, leaving behind their young children in a safe and secure care-environment (Ardington, Case and Hosegood 2009; Hosegood and Timæus 2005). Currently in South Africa, in addition to means-testing, only those aged 60+ qualify for the old-age pension of around R1,200 per month (approximately US $145). This amount is lower than the 2008 average income for South Africa of R1,456, although above the average income of R816 for Blacks – the dominant race group in rural South Africa (Leibbrandt, Finn and Woolard 2012). As such, old-age pension grants to persons aged 60+ have become the main source of household income in rural South Africa (Booysen 2004; Kimuna and Makiwane 2007).

The psycho-social ability of older people to provide care, particularly due to the impact of HIV on their children and grandchildren, has been previously explored (Boon *et al.* 2010; Schatz and Ogunmefun 2007). Others have also explored the secondary consequences that older people experience as a result of caring for HIV-infected adults and children, such as isolation and separation from family (Hosegood *et al.* 2007; Ogunmefun, Gilbert and Schatz 2011). Government cash transfers in South Africa have helped mitigate the financial implications of care-giving by older people (Ardington *et al*. 2010). However, in contrast to studies of the psycho-social and financial challenges of care-giving by older people, far less is known about the relationship between care-giving and care-receipt and the functional ability (physical health) and emotional wellbeing of older people infected or affected by HIV in rural South Africa.

Chepngeno-Langat *et al*. (2011) investigated the association between care-giving to people living with AIDS and poor health among older care-givers aged 50+ living in two Nairobi slums, based on self-reported health using the World Health Organization Disability Assessment Schedule (WHODAS) and the presence of a severe health problem. While no significant differences were observed between female AIDS care-givers and female non-care-givers, male AIDS caregivers were significantly more likely to report disability and have a severe health problem compared with male non-care-givers. In another of the few available studies, qualitative data were used to assess the impact on health and wellbeing of older care-givers in rural Uganda (Ssengonzi 2009). Care-giving was shown to increase the likelihood of emotional, physical and psychological stress (Ssengonzi 2009). However, in another study from east Africa that used three waves of panel data collected between 2005 and 2007 (Ice *et al.* 2010), care-giving was not associated with ill-health as objectively measured by body mass index, blood pressure, haemoglobin and blood glucose levels. Over time older people who were primary care-givers to orphaned children did have poorer self-perceived health status and mental health (Ice *et al*. 2010). A recent study in which the burden of care-giving to orphaned and non-orphaned children was compared showed older care-givers to orphans were more likely to be caring for a higher number of children, more likely to be caring for children with mental or behavioural problems, less likely to receive adult help, and to have more chronic illnesses and generally poorer health (Govender *et al.* 2012).

Understanding how care-giving influences health and wellbeing is essential to supporting older people in rural South Africa who are increasingly taking on the responsibilities of caring for their adult offspring and grandchildren. The aim of this paper is to examine whether care-giving to adults and/or to children by HIV-infected or -affected older people in rural South Africa is associated with poor functional disability and emotional wellbeing. The research also aims to assess the associated effect of older people *receiving* care on their health and emotional wellbeing. Older people are defined as persons aged 50 years and above, in line with previous ageing work in Africa (Hosegood and Timæus 2006; Kowal *et al.* 2010; Ssengonzi 2007); whereas adults are defined as persons aged 18–49 years and children are those aged under 18 years.

## Methods

### Sources of data and study design

Data used for this analysis came from the cross-sectional Health and Well-being of Older People Study (WOPS), whose main aim was to investigate the direct and indirect effects of HIV on the health and wellbeing of older people aged 50 years and over within a surveillance population located in north-eastern KwaZulu-Natal, South Africa. Covering a 435 square kilometres area, the Africa Centre demographic and health surveillance has collected birth, death and migration data on approximately 90,000 household members since 2000, and sexual behaviour, general health and HIV status data on all adults aged 15 years and over since 2003. In addition, information on all HIV-infected persons accessing care at the hospital or any one of the 17 primary health-care clinics in the Hlabisa sub-district (six of which are within the surveillance area) is collected in the Antiretroviral Therapy Evaluation and Monitoring Information System (ARTemis). The ARTemis database is hosted at the Africa Centre and with ethical approval we have been able to link the 40 per cent of people in ARTemis resident in the surveillance area to the Africa Centre surveillance information (Houlihan *et al.* 2011). Detailed information about the Africa Centre's surveillance can be found in earlier publications (Hosegood, Benzler and Solarsh 2006; Tanser *et al.* 2007) and by visiting www.africacentre.com.

WOPS data and the main findings regarding the health status and emotional wellbeing of study participants are documented in detail in Nyirenda *et al.* (2012). In sum, a shortened version of the World Health Organization (WHO) Study on Global Ageing and Adult Health (SAGE) instrument (WHO 2011) was used to collect the data in this WHO-supported study. Conducted between March and August 2010, the main criteria for inclusion was being aged 50+, and under observation and resident within the Africa Centre surveillance. Stratified random sampling was used to select participants into four specified strata:

• Stratum 1 participants had to be HIV-infected and receiving HIV treatment for a year or longer.
• Stratum 2 participants had to be HIV-infected and receiving HIV treatment for three months or less or waiting to initiate treatment.
• Stratum 3 consisted of participants who had an adult offspring (18–49 years) who was HIV-infected and receiving treatment for a year or longer, or receiving treatment for three months or less.
• Stratum 4 was composed of participants who had experienced the death of an adult household member between 2008 and 2010 (two years prior to the study), which was classified as HIV-related from the verbal autopsy data.

For this analysis, participants in strata 1 and 2 were categorised as ‘HIV-infected’, while those in strata 3 and 4 were categorised as ‘HIV-affected’. The target total sample size was 400 older people which power calculations showed to be adequate to test for statistically significant differences at the 5 per cent level of significance between the groups. The first stage of the sampling was linking all persons aged 50+ in the surveillance to ARTemis and then identifying all eligible persons for this study as per inclusion criteria stated earlier. There were 241, 117, 662 and 142 eligible participants for strata 1–4, respectively, from which we randomly selected 100 participants for each group. These were visited at their homesteads and enrolled into the study. All eligible individuals found at a visited household were invited to participate in the study. Persons too sick to participate (N=3), non-contacts (N=2) and those who refused participation (N=4) were excluded; in these cases replacements were selected from the respective eligible population. Our consent level was high because we targeted individuals that were already actively participating in the Africa Centre surveillance system. The final sample size was 422 individuals because in some households more than one person met the inclusion criteria.

### Dependent variables

The two principal dependent variables were physical functional ability health status and quality of life, a measure of emotional wellbeing, both derived using validated WHO instruments (WHO 2010; WHOQoL Group 1993). We also explored self-rated overall health status, reported health status in the two weeks before the interview and self-reported quality of life as other measures of health and wellbeing.

### Physical functional ability health status

This was measured using the WHODAS (WHO 2010). Participants in WOPS were asked about difficulties experienced in the last 30 days with performing activities of daily living such as walking, standing, stooping, kneeling or crouching, getting up from a sitting position, getting up from a lying down position, picking up things from the table, doing household chores, as well as instrumental activities of daily living like getting dressed, bathing, eating, getting to the toilet, using public transport and participation in community activities. Responses to these items were based on a five-point Likert-type scale of ‘none’, ‘mild’, ‘moderate’, ‘severe’ and ‘extreme/cannot do’. Responses to these items were used to compute a WHODAS disability score ranging from 0 to 100. Where 0 represents excellent functional ability and 100 indicates extreme difficulties in performing physical activities (low functional ability). To make interpretation easier we took an inverse of the score (WHODASi), such that now a low score meant low functional ability and a high score meant high functional ability. The score was further divided into quintiles, which were in turn categorised into a dichotomous physical health status variable for use in the logistic regressions. The category ‘poor health’ comprised participants in health quintiles 1–3, while ‘good health’ category was made up of participants in quintiles 4 and 5. This was done for comparability with similar earlier work (Ng *et al.* 2010; Xavier Gomez-Olive *et al.* 2010).

### Quality of life

Subjective or emotional wellbeing was measured using the WHO Quality of Life (WHOQoL) score (WHOQoL Group 1993). A score is assigned to each person based on their answers to questions about satisfaction with, among other things, their self, health, living conditions, personal relationships, ability to perform daily living activities and their life as a whole. Also included were questions on how often they felt unable to control important things in their life and to cope with situations. Eight questions were used to compute the WHOQoL score, which ranged from 8 to 40. This was then transformed into a scale of 0–100, where 100 corresponded to best quality of life (best emotional wellbeing). For the logistic regressions, as for physical health status, quintiles and then a dichotomous variable of quality of life (‘poor’ compared to ‘good’) were created from the WHOQoL score. Details about the WHODAS and WHOQoL have been described elsewhere (Nyirenda *et al*. 2012).

### Self-rated health status

Participants in WOPS were asked ‘In general, how would you rate your health today?’ In spite of some comparability and inconsistency concerns (Bowling 2005; Fayers and Sprangers 2002; Salomon *et al.* 2009), this global question of self-reported health status has been shown to be a good indicator of public health and mortality in a population even across different cultures (Fayers and Sprangers 2002; Idler and Benyamini 1997; Wang *et al.* 2008). In WOPS the question had five response categories (‘very good’, ‘good’, ‘moderate’, ‘bad’ and ‘very bad’). Following an approach adopted by others (Debpuur *et al.* 2010), the response categories were collapsed into two: ‘Good’ composed of ‘very good’ and ‘good’; and ‘Bad’ comprised of the categories ‘moderate’, ‘bad’ and ‘very bad’.

### Health status in the last two weeks

Respondents in WOPS were asked how their health had been in the two weeks prior to the interview date. From the five-point Likert-type scale of the question, a dichotomous variable was created: ‘Good’ (‘very good’ and ‘good’) and ‘Bad’ (‘moderate’, ‘bad’ and ‘very bad’). This question may be a better indicator of health status than the global self-rated health status question which only collects data on health status on the day of contact, whereas this question allows the respondent to reflect on their health over a two-week period, and hence is likely to capture more episodes of ill-health (or a longer duration of ill-health) in older people compared to the global question described above which refers to a single day.

### Self-reported quality of life

Participants in WOPS were asked ‘How would you rate your overall quality of life?’ This question again had five response categories. These were, as above, created into a dichotomous variable of ‘good’ and ‘bad’ quality of life. This question was included as an outcome variable to compare with outcomes from the WHOQoL measure.

### Independent variables: care-giving and care-receiving

Care-giving was defined as an older person assisting an adult (18–49) or child (<18 years) with activities of daily living. Care was further split into physical and nursing care. The former referred to assistance with activities such as cooking, fetching water, taking to the clinic or traditional healer, shopping and moving around, while the later referred to assistance with eating, bathing, dressing, toileting, administering medicines and dressing of wounds. When older persons themselves were unable to perform any of these tasks and were getting assistance from someone else, they were defined as ‘care-receiving’. The main independent variables care-giving and care-receiving are initially treated separately as dichotomous variables: ‘Yes, providing (or receiving) care’ or ‘No, not providing (or receiving) care’. Later care-giving and care-receiving is combined into an overall ‘Care’ variable with four categories: ‘Neither care-giver nor care-receiver’, ‘Care-giver only’, ‘Care-receiver only’ and ‘Both care-giver and care-receiver’.

We also asked participants about provision and receipt of financial assistance with regard to paying for food, clothing, doctor or traditional healer fees, paying for transportation and school fees for children (<18 years), but this was considered separately from care as providing physical or nursing care is likely to have very different effects on health and wellbeing from financial assistance.

### Other control variables

Given that previous research has shown that the health and wellbeing of older people is affected by different socio-demographic, economic and household living-arrangement factors, we controlled for several of these factors in order to arrive at the independent effect of ‘care’ on the health and emotional wellbeing of older people. The factors controlled for were: age (50–59, 60–69, 70–79 and 80+ years), sex (male or female), marital status (never married, currently married, previously married), education attainment (no formal education, primary, secondary), receipt of government grants, household living arrangements (household headship, household composition (older person-only, living with children only or living with both adults and children)) and household socio-economic status (household wealth quintiles and self-perceived household financial status).

### Data analysis

This analysis is based on all 422 persons aged 50 years and above who participated in WOPS, which yielded detailed information on older people's health status and care patterns. Initially, the data were analysed using descriptive and bivariate analyses to describe the socio-demographic and living arrangements of these older people. Then, multivariable logistic regressions were used to examine the association of care-giving to adults (18–49 years) and to children (<18 years) with physical functioning health status, emotional wellbeing, health in the last two weeks, overall self-rated health status and self-reported quality of life, controlling for socio-demographic and living arrangements variables. Additionally, we assessed the relationship between receipt of care by older people and their health and wellbeing. All data were analysed using Stata IC 11.2 (StataCorp 2009).

## Results

### Socio-demographic and economic characteristics study population

Overall, the WOPS sample was predominantly female (75 per cent), the majority (45 per cent) were aged 50–59 years, predominantly not working (91 per cent), and nearly half were currently married and had no formal education. Most were household heads or belonged to households headed by their spouse. When asked about their financial situation at interview date compared to an arbitrary chosen three years ago referent point, 52 per cent rated their situation as worse off, 16 per cent said they were more comfortable now than three years ago, while 32 per cent said their financial situation had not changed. Over 80 per cent of study participants were in receipt of government cash transfers (old-age pension grant, 54 per cent; disability grant, 27 per cent) (Table 1).

**Table 1.** Socio-demographic characteristics by care-giving status, rural South Africa, 2010

Characteristics	Overall	Non-care-giver	Care-giver	*p*
	*Percentages (N)*			
	100 (422)	37 (156)	63 (266)	
Sex:				<0.001
Male	25.1 (106)	57.5 (61)	42.5 (45)	
Female	74.9 (316)	30.1 (95)	69.9 (221)	
Age group:				0.188
50–59	44.5 (188)	34.0 (64)	66.0 (124)	
60–69	30.3 (128)	35.2 (45)	64.8 (83)	
70+	25.1 (106)	44.3 (47)	55.7 (59)	
Marital status:				<0.001
Never married	27.7 (117)	41.0 (48)	59.0 (69)	
Married	48.8 (206)	46.1 (95)	53.9 (111)	
Previously married	23.5 (99)	13.1 (13)	86.9 (86)	
Education:				0.959
NFE/AEO	47.6 (201)	36.3 (73)	63.7 (128)	
⩽6 years	33.2 (140)	37.9 (53)	62.1 (87)	
>6 years	19.2 (81)	37.0 (30)	63.0 (51)	
Employment status:				0.489
Not working	90.5 (382)	37.2 (142)	62.8 (240)	
Working	9.0 (38)	34.2 (13)	65.8 (25)	
Missing	0.5 (2)	50.0 (1)	50.0 (1)	
Grant receipt:				0.081
None	19.4 (82)	26.8 (22)	73.2 (60)	
Disability	27.0 (114)	42.1 (48)	57.9 (66)	
Old-age pension	53.6 (226)	38.1 (86)	61.9 (140)	
Perceived financial status:				0.001
Better	15.9 (67)	56.7 (38)	43.3 (29)	
No change	32.3 (136)	33.8 (46)	66.2 (90)	
Worse	51.9 (219)	32.9 (72)	67.1 (147)	
Household headship:				0.201
Self	57.8 (244)	39.3 (96)	60.7 (148)	
Spouse	20.6 (87)	28.7 (25)	71.3 (62)	
Other	21.6 (91)	38.5 (35)	61.5 (56)	
Wealth quintile:				0.515
First	17.5 (74)	41.9 (31)	58.1 (43)	
Second	21.8 (92)	39.1 (36)	60.9 (56)	
Third	19.4 (82)	28.0 (23)	72.0 (59)	
Fourth	16.4 (69)	36.2 (25)	63.8 (44)	
Fifth	12.6 (53)	41.5 (22)	58.5 (31)	
Missing	12.3 (52)	36.5 (19)	63.5 (33)	
Household typology:				0.006
Solo household	2.1 (9)	77.8 (7)	22.2 (2)	
Older-only	1.7 (7)	57.1 (4)	42.9 (3)	
Skip-generation	0.9 (4)	50.0 (2)	50.0 (2)	
Second-generation	4.7 (20)	65.0 (13)	35.0 (7)	
Multi-generation	86.7 (366)	33.9 (124)	66.1 (242)	
Missing	3.8 (16)	37.5 (6)	62.5 (10)	

*Notes*: The *p* value compares non-care-givers to care-givers by socio-demographic characteristics. The ‘Overall’ column gives column percentages of the total sample (N=422). For ‘Non-care-giver’ and ‘Care-giver’ columns the percentages are row-wise within each category of the socio-demographic characteristic. NFE stands for No Formal Education, while AEO stands for Adult Education Only (these are special classes designed to teach adults who have no formal education some basic literacy and numeracy).

In total, 63 per cent of study participants were providing care to either an adult or child (Table 1). Significant gender differences were observed with regard to care-giving; over two-thirds of older women were care-givers compared to less than half among men. We also found a substantially higher proportion of previously married (separated, divorced or widowed) older people to be care-givers. Other significant differences with regard to care-giving status and socio-demographic characteristics were observed for self-reported change in financial situation and household typology (Table 1).

### Characteristics of care-giving to adults or children by older people in rural South Africa

Further analyses (not shown) of care-giving to adults (18–49 years) and to children (<18 years) revealed that in total just under 42 per cent (N=175) of WOPS participants were providing care to adult household members (42.1 per cent among women, 39.6 per cent among men); while 57 per cent (N=239) were providing care to at least one child. There were significant gender differences in care-giving to children; 63.9 per cent of women compared to 34.9 per cent of men were care-givers to children. Although most of the participants perceived their financial situation to be worse off than three years earlier, a higher proportion felt this way among older people providing care to children compared to care-givers to adults (59% *versus* 47%). Most of the care among care-givers to adults was physical care only (93 per cent). Only a minority (3%, N=13) of older people were providing adults with nursing care. Among care-providers to children, 63 per cent were providing children with both physical and nursing care; 37 per cent were providing physical care only.

Over 27 per cent of adults needing care were said to be doing so for HIV-related reasons. Of the 175 older people providing care to adults, 42 per cent reported that the adults cared for had been contributing to household income prior to their illness. When asked whether they had difficulties in providing care to adults, 65 per cent (N=114) said they found it very difficult and a further 23 per cent said they had some difficulty. Approximately 10 per cent said they had no difficulties. Roughly 4 per cent of children were reported to be needing care for HIV-related reasons. Among older people providing care to children (N=239), only 23 per cent of the children being cared for were contributing to household income before, and 48 per cent of the older people reported experiencing great difficulties in providing care to children. Although we stratified the analysis to consider care-giving to adults and children separately, care-giving to adults and children is by no means mutually exclusive; of the care-giving participants, over 56 per cent (N=148) were caring for both adults and children.

Adjusting for age, sex, marital status, education, grant receipt, household headship and household wealth status, older people who were HIV-infected were significantly less likely than HIV-affected participants to be giving care to adults (adjusted odds ratio (aOR)=0.52, *p*=0.02) or to children (aOR=0.39, *p*<0.001). Women were significantly more likely to be care-providers for adults or children (aOR=2.74, *p*<0.001). Other factors significantly associated with care-giving to both adults and children in adjusted logistic models were marital status and self-perceived financial status as not having changed or gotten worse compared to three years ago.

### Care-receiving characteristics and patterns among older people in rural South Africa

A very high proportion of WOPS participants (84%, N=356) reported receiving care. Fetching water was the main activity (N=329) older people were being assisted with. Grandchild (65%), children (64%) and spouse (18%) were the main sources of physical assistance or care. Approximately 5 per cent said they were receiving care from a son or daughter-in-law or from own siblings. Outside of the family (children and grandchildren), just over 2 per cent of older people had hired assistants (primarily domestic workers) to help with activities of daily living and roughly 1 per cent received care from neighbours or community volunteers.

With regard to nursing care, which comprises assistance with bathing, eating, toileting, incontinence and taking medicines, only 6 per cent (N=25) of older people reported they were receiving such assistance. This is a very small fraction of the 265 (63%) who said they were in need of care, support or treatment. HIV- and tuberculosis-related (N=162) and health-related but not HIV (N=69) were the main reasons reported for being in need of care, support or treatment. Around 60 per cent (N=208) of older people receiving physical or nursing care reported that they were satisfied with the care received, while 35 per cent (N=125) said they were dissatisfied.

Financial assistance was another area of need for older people in rural South Africa. Items that older people in the study reported being in need of financial support for included: food (89%), clothing (88%) and transportation (83%). The government was reported by study participants as the main source of financial assistance, reflecting the fact that 81 per cent of the sample were in receipt of either old-age pension or disability grant. In a distant second and third, respectively, as sources of financial support, were son or daughter (8%) and spouse (7%). Over 91 per cent (N=304) of grant recipients said they used the grant for household expenses (such as food, school and health-care needs of other household members), only 8 per cent (N=28) used the grant received for their own upkeep (*e.g.* buying own clothes, accessories, books and own health-care needs). As testament to how widely and easily accessible the grants are, among grant recipients (N=340) the majority (66%) said it had not been difficult for them to start receiving this financial assistance; 15 per cent said it had been a little difficult and 17 per cent said it was very difficult. Around 78 per cent (N=329) of older people said they had been contributing to household income in the past before being in poor health, with 64 per cent (N=211) of these saying they had been the main income provider.

Adjusting for sex, age, marital status, education, receipt of grants, household headship and household wealth, older people who were HIV-infected relative to those HIV-affected were less likely to be care-receivers (aOR=0.32, *p*<0.01). Older people who had six years or less of education compared to no formal education were similarly less likely to be receiving care (aOR=0.45, *p*=0.03). On the other hand, self-rating of financial status today compared to three years ago as ‘no change’ (aOR=3.40, *p*=0.01) or as ‘worse’ relative to better was associated with significantly higher odds of being in receipt of care in adjusted logistic analyses (aOR=2.92, *p*=0.01).

### Being a care-giver and care-recipient simultaneously and associated factors

Table 2 presents correlates of care, with *p*-values showing chi-square differences between participants who were exclusively care-givers and those who were care-receivers only. About 56 per cent of study participants were simultaneously care-givers and care-receivers, while 9 per cent were neither care-givers nor care-receivers. Statistically significant differences (at *p*<0.001) between care-giver only and care-receiver only participants were observed for all correlates considered except employment status, household headship and household wealth quintiles (Table 2).

**Table 2.** Proportion giving and/or receiving care, rural South Africa, 2010

Characteristics	Neither	Care-giver only	Care-receiver only	Both	*p*
	*Percentages (N)*				
	8.8 (37)	6.6 (28)	28.2 (119)	56.4 (238)	
Sex:					<0.001
Male	13.2 (14)	2.8 (3)	44.3 (47)	39.6 (42)	
Female	7.3 (23)	7.9 (25)	22.8 (72)	62.0 (196)	
Age group:					<0.001
50–59	11.7 (22)	11.7 (22)	22.3 (42)	54.3 (102)	
60–69	8.6 (11)	4.7 (6)	26.6 (34)	60.2 (77)	
70+	3.8 (4)	0 (0)	40.6 (43)	55.7 (59)	
Marital status:					<0.001
Never married	16.2 (19)	7.7 (9)	24.8 (29)	51.3 (60)	
Married	6.3 (13)	6.8 (14)	39.8 (82)	47.1 (97)	
Previously married	5.1 (5)	5.1 (5)	8.1 (8)	81.8 (81)	
Education:					0.027
NFE/AEO	6.0 (12)	3.0 (6)	30.3 (61)	60.7 (122)	
⩽6 years	11.4 (16)	8.6 (12)	26.4 (37)	53.6 (75)	
>6 years	11.1 (9)	12.3 (10)	25.9 (21)	50.6 (41)	
Employment:					0.622
Employed	8.1 (31)	6.5 (25)	29.1 (111)	56.3 (215)	
Unemployed	15.8 (6)	7.9 (3)	18.4 (7)	57.9 (22)	
Grant receipt:					<0.001
None	11.0 (9)	11.0 (9)	15.9 (13)	62.2 (51)	
Disability	14.0 (16)	11.4 (13)	28.1 (32)	46.5 (53)	
Old-age pension	5.3 (12)	2.7 (6)	32.7 (74)	59.3 (134)	
Financial status self:					0.001
Better	16.4 (11)	10.4 (7)	40.3 (27)	32.8 (22)	
No change	5.9 (8)	3.7 (5)	27.9 (38)	62.5 (85)	
Worse	8.2 (18)	7.3 (16)	24.7 (54)	59.8 (131)	
Household headship:					0.31
Self	9.8 (24)	6.1 (15)	29.5 (72)	54.5 (133)	
Spouse	3.4 (3)	10.3 (9)	25.3 (22)	60.9 (53)	
Other	11.0 (10)	4.4 (4)	27.5 (25)	57.1 (52)	
Wealth quintile:					0.782
First	6.8 (5)	8.1 (6)	35.1 (26)	50.0 (37)	
Second	9.8 (9)	7.6 (7)	29.3 (27)	53.3 (49)	
Third	8.5 (7)	3.7 (3)	19.5 (16)	68.3 (56)	
Fourth	8.7 (6)	5.8 (4)	27.5 (19)	58.0 (40)	
Fifth	13.2 (7)	7.5 (4)	28.3 (15)	50.9 (27)	
Missing	5.8 (3)	7.7 (4)	30.8 (16)	55.8 (29)	
Household typology:					<0.001
Solo household	55.6 (5)	11.1 (1)	22.2 (2)	11.1 (1)	
Older-only	0 (0)	14.3 (1)	57.1 (4)	28.6 (2)	
Skip-generation	25.0 (1)	0 (0)	25.0 (1)	50.0 (2)	
Second-generation	20.0 (4)	10.0 (2)	45.0 (9)	25.0 (5)	
Multi-generation	6.8 (25)	6.6 (24)	27.0 (99)	59.6 (218)	
Missing	12.5 (2)	0 (0)	25.0 (4)	62.5 (10)	

*Notes*: The *p* value is for chi-square comparison of care-givers to care-receivers by socio-demographic characteristics. NFE stands for No Formal Education, while AEO stands for Adult Education Only (these are special classes designed to teach adults who have no formal education some basic literacy and numeracy).

In logistic regression analyses (data not shown) allowing for age, marital status, education, receipt of grants, household headship and household wealth, women were nearly twice as likely to simultaneously be giving and receiving care (aOR=1.95, *p*=0.01). By marital status, participants who had been previously married were five times more likely to be care-givers and care-receivers than those never married (aOR=5.01, *p*<0.001). Other factors significantly associated with simultaneous giving and receiving care were a worsening financial situation (aOR=2.78, *p*<0.01) and belonging to a medium household wealth quintile (aOR=2.42, *p=*0.02). In contrast, older people who were HIV-infected were less likely to be simultaneously giving and receiving care than HIV-affected participants (aOR=0.45, *p*<0.001).

### Effects of care-giving to adults on the health and wellbeing of older people

Table 3 shows the distribution of participants in good or poor health by the selected health measures. Chi-square tests showed statistically significant differences in functional ability (WHODAS) and emotional wellbeing (WHOQoL). Among non-care-givers, the majority (68.6%) were in poor physical functioning health. In contrast, most care-givers were in poor emotional wellbeing (68.8%).

**Table 3.** Health status by care-giving status among older people, rural South Africa, 2010

Characteristics	Overall	Non-care-giver	Care-giver	*p*
	*Percentages (N)*			
	100 (422)	37 (156)	63 (266)	
Physical functioning (WHODAS):				0.011
Good	39.3 (166)	31.4 (49)	44.0 (117)	
Poor	60.7 (256)	68.6 (107)	56.0 (149)	
Quality of life (WHOQoL):				0.001
Good	37.2 (157)	47.4 (74)	31.2 (83)	
Poor	62.8 (265)	52.6 (82)	68.8 (183)	
Self-rated overall health status:				0.738
Good	45.7 (193)	46.8 (73)	45.1 (120)	
Poor	54.3 (229)	53.2 (83)	54.9 (146)	
Health last two weeks:				0.119
Good	22.7 (96)	18.6 (29)	25.2 (67)	
Poor	77.3 (326)	81.4 (127)	74.8 (199)	
Self-reported quality of life:				0.133
Good	15.2 (64)	18.6 (29)	13.2 (35)	
Poor	84.8 (358)	81.4 (127)	86.8 (231)	

*Notes*: The *p* value is for chi-square comparison of non-care-givers to care-givers by health measure. WHODAS: World Health Organization Disability Assessment Schedule. WHOQoL: World Health Organization Quality of Life.

**Figure 1.**
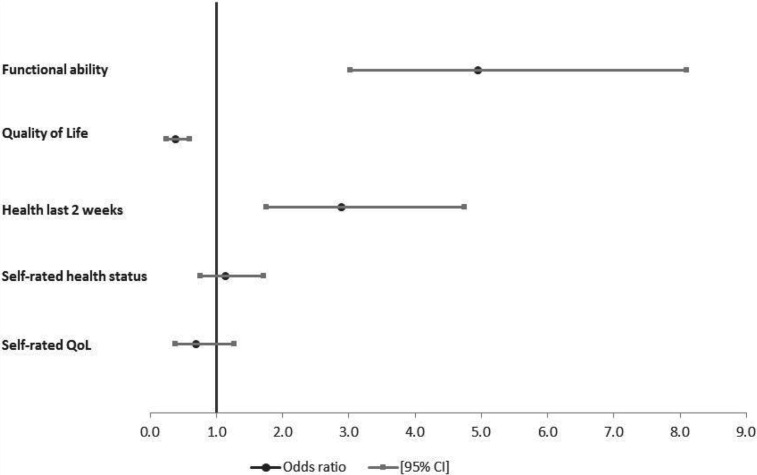
Odds of being in good health by health measure for older people giving care to adults, South Africa, 2010.

*Notes*: Odds ratios adjusted for age, gender, marital status, education, receipt of government grants, household wealth quintiles and HIV status (HIV-infected *versus* HIV-affected). QoL: quality of life. CI: confidence interval.

Logistic regressions were used to assess the association of care-giving to adults and/or to children relative to non-care-givers with the health and wellbeing of older people, adjusting for HIV state (HIV-infected *versus* HIV-affected), age, gender, marital status, education level completed, receipt of government grants and household wealth status. Results of the regression analyses of the association between care-giving to adults and health status are presented in Figure 1. Study participants who were providing care to adults were more likely to have good functional ability relative to older people not providing care (*p*<0.001) (Figure 1). Older people who were providing care to adults were similarly more likely to report themselves as being in good functional ability in the last two weeks (*p*<0.001) and in the overall self-rated health status question (*p*=0.53). The association of care-giving to adults with quality of life (emotional wellbeing) was stronger than that of the relationship between care-giving and functional ability. Older people who were care-givers to adults had 62 per cent lower likelihood of having good quality of life (*p*<0.001), adjusting for the same factors as in the functional ability models (Figure 1). Although statistical significance was not reached, study participants who were care-givers to adults were similarly less likely to rate their own quality of life as good (*p*=0.24).

In stratified analyses by HIV status, similar patterns in the effect of care-giving to adults or to children on older people's health and wellbeing were observed (Table 4). In both HIV-infected and HIV-affected participants, older people who were care-givers were statistically significantly more likely to be in good functional ability or to rate their health in the past two weeks as good relative to non-care-givers. However, care-givers were less likely to have a good quality of life (WHOQoL) and self-reported quality of life, although results were only statistically significant for quality of life (WHOQoL) among care-givers to adults.

**Table 4.** Effect of care-giving or -receiving on older people's health status by HIV status

Dependent variables	Odds of being in good health for older care-givers to adults		Odds of being in good health for older care-givers to children		Odds of being in good health for older people receiving care	
	HIV-infected	HIV-affected	HIV-infected	HIV-affected	HIV-infected	HIV-affected
	*Adjusted odds ratio (95% confidence intervals)*					
Functional ability (WHODAS):						
Poor	1.00	1.00	1.00	1.00	1.00	1.00
Good	3.15 (1.57–6.33)	8.39 (3.91–17.98)	2.00 (0.97–4.14)	3.27 (1.55–6.92)	0.38 (0.17–0.85)	1.04 (0.27–3.95)
Constant (−2 LL)	111.21**	101.12**	114.92**	113.80**	111.21**	101.12**
Model *χ*^2^	58.76	69.11	51.35	43.75	58.76	69.11
df	13	13	13	13	13	13
N	203	219	203	219	203	219
Quality of life (WHOQoL):						
Poor	1.00	1.00	1.00	1.00	1.00	1.00
Good	0.38 (0.20–0.73)	0.41 (0.21–0.80)	0.83 (0.43–1.59)	0.57 (0.29–1.10)	0.61 (0.28–1.31)	0.43 (0.12–1.51)
Constant (−2 LL)	124.50*	117.19**	128.79	119.32**	124.50*	117.19**
Model *χ*^2^	27.67	41.56	19.08	37.3	27.67	41.56
df	13	13	13	13	13	13
N	203	219	203	219	203	219
Health status last two weeks:						
Poor	1.00	1.00	1.00	1.00	1.00	1.00
Good	2.17 (1.06–4.45)	4.31 (2.00–9.33)	2.44 (1.07–5.57)	1.78 (0.83–3.83)	0.98 (0.43–2.24)	2.36 (0.28–20.04)
Constant (−2 LL)	102.03*	98.06*	101.87*	104.65	102.03*	98.06*
Model *χ*^2^	22.58	28.95	22.9	15.84	22.58	28.95
df	13	13	13	13	13	13
N	203	219	203	219	203	219
Self-rated overall health status:						
Poor	1.00	1.00	1.00	1.00	1.00	1.00
Good	1.39 (0.75–2.57)	0.97 (0.54–1.74)	1.29 (0.67–2.48)	0.96 (0.52–1.76)	0.75 (0.37–1.54)	0.43 (0.13–1.42)
Constant (−2 LL)	131.85	138.52*	132.12	138.52*	131.85	138.52*
Model *χ*^2^	17.32	23.21	16.78	23.22	17.32	23.21
df	13	13	13	13	13	13
N	203	219	203	219	203	219
Self-reported quality of life:						
Poor	1.00	1.00	1.00	1.00	1.00	1.00
Good	0.70 (0.32–1.55)	0.70 (0.25–1.96)	0.58 (0.25–1.32)	1.22 (0.42–3.52)	0.40 (0.17–0.93)	1.31 (0.11–15.98)
Constant (−2 LL)	92.74*	55.60*	92.27*	55.76**	92.74*	55.60*
Model *χ*^2^	24.16	24.05	25.1	23.73	24.16	24.05
df	13	12	13	12	13	12
N	203	204	203	204	203	204

*Notes*: Logistic regressions were run separately for each outcome variable. In each model the following were controlled for: age, sex, education, receipt of government grants and household wealth quintiles. WHODAS: World Health Organization Disability Assessment Schedule. WHOQoL: World Health Organization Quality of Life. LL: log likelihood. df: degrees of freedom.

*Significance levels*: * *p*<0.05, ** *p*<0.001.

### Effects of care-giving to children on the health and wellbeing of older people

**Figure 2.**
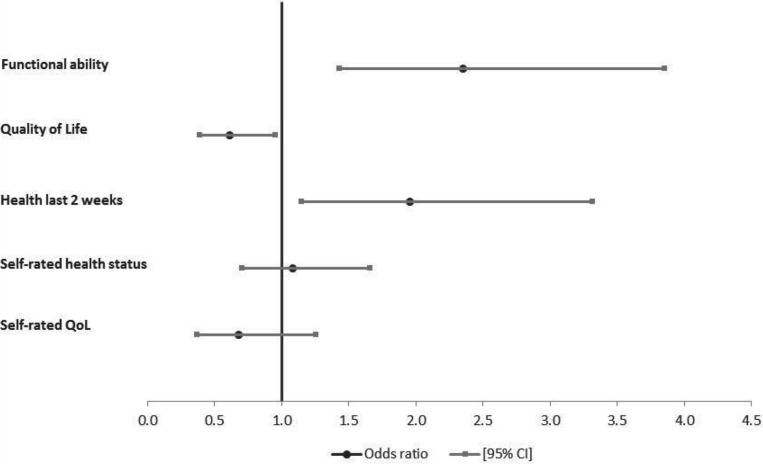
Odds of being in good health by health measure for older people giving care to children, South Africa, 2010.

*Notes*: Odds ratios adjusted for age, gender, marital status, education, receipt of government grants, household wealth quintiles and HIV status (HIV-infected *versus* HIV-affected). QoL: quality of life. CI: confidence interval.

The associations of care-giving to children with respective health measures are shown in Figure 2; findings were similar to those for care-giving to adults. Care-giving to children was significantly associated with higher chances of being in good functional ability (*p*<0.001) and good health status in the last two weeks (*p*=0.01). But care-giving was strongly associated with lower chances of being in good quality of life (*p*=0.03), adjusting for HIV state, age, gender, marital status, education level completed, receipt of government grants and household wealth status (Figure 2). There was no evidence to support a significant association of self-rated overall health status (*p*=0.72) and self-reported quality of life (*p*=0.22) with care-giving to children. In stratified analyses by HIV status (results not shown), care-giving to children was only significantly associated with higher odds of being in good functional ability among HIV-affected participants (aOR=3.27, *p*<0.001) and in good health in the last two weeks among HIV-infected participants (aOR=2.44, *p*=0.03).

Among the sub-sample of older people providing care to both adults and children, in multivariable models care-giving was associated with higher odds of being in good functional ability (aOR=5.74, *p*<0.001), good health status in the last two weeks (aOR=3.15, *p*<0.001) and self-rated overall health status (aOR=1.15, *p*=0.57). In contrast, it was associated with less likelihood of having a good quality of life (aOR=0.36, *p*<0.001) and good self-reported quality of life (aOR=0.58, *p*=0.15).

### Association of care-receiving with health status and emotional wellbeing of older people

**Figure 3.**
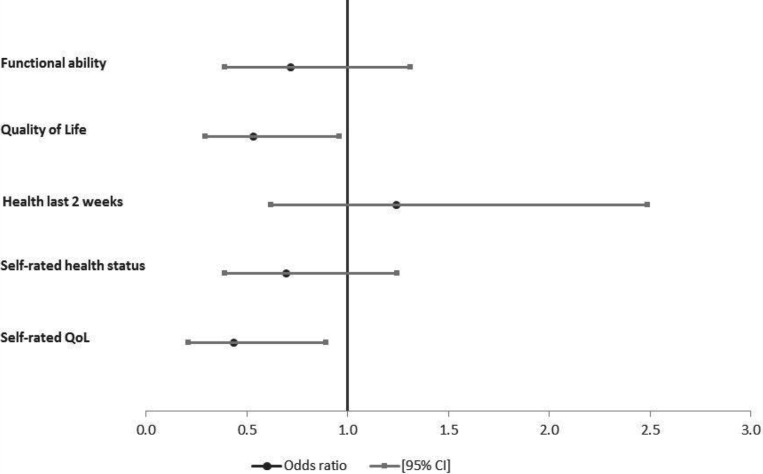
Odds of being in good health by health measure for older people receiving care, rural South Africa, 2010.

*Notes*: Odds ratios adjusted for age, gender, marital status, education, receipt of government grants, household wealth quintiles and HIV status (HIV-infected *versus* HIV-affected). QoL: quality of life. CI: confidence interval.

As expected, older people who were receiving care were less likely to be in good health on all health measures considered except for self-reported health status in the last two weeks in adjusted multivariable models (Figure 3). Factors adjusted for were HIV status, age, gender, marital status, education level completed, receipt of government grants and household wealth quintiles. However, only quality of life (WHOQoL) (aOR=0.53, *p*=0.04) and self-reported quality of life (aOR=0.43, 0.02) were statistically significantly associated with lower odds of being in good health for participants in receipt of care relative to those not receiving care. In separate analyses of HIV-infected and HIV-affected participants (Table 4), only functional ability and self-reported quality of life in HIV-infected participants were statistically significantly associated with less likelihood of older people in receipt of care being in good health.

Older people who were care-givers and care-receivers at the same time had over two-fold higher likelihood of being in good functional ability (*p*<0.001), but had about 47 per cent lower chances of enjoying a good quality of life (*p*=0.005). The association of health in the last two weeks with being a care-giver and care-receiver simultaneously was borderline statistically significant (*p*=0.051), but self-rated health status (*p=*0.79) and self-reported quality of life (*p*=0.16) were not statistically significant.

## Discussion

In our cross-sectional study, we found a very high level of care-giving among older people in this rural community. More than two in five older people were care-givers to adults and three in five provided care to children, mainly grandchildren. Overall, over 60 per cent of the study participants were care-givers to at least one adult or child; of these over one-half were care-givers to both adults and children co-resident in their household. Whereas about 30 per cent of care-giving to adults was associated with HIV infection, much of the care-giving to children was not because of ill-health but reflected their dependence due to young age. Our findings further suggest care-givers among both HIV-infected and -affected older people were more likely to be in good physical health than non-care-givers. On the other hand, care-giving particularly to adults was associated with a higher likelihood of reporting a poor quality of life (or poor emotional wellbeing). Similarly, older people who were simultaneously care-givers and -receivers had good functional ability but were half as likely as the remainder of the study participants to report good quality of life.

A higher proportion of women than men were care-givers (70% compared to 42%). Previous findings from the same study population shows that women are more likely to report poor physical and emotional wellbeing than men (Nyirenda *et al.* 2012) irrespective of HIV status, which is consistent with findings from elsewhere that women tend to report more ill-health, disability and mental health problems than men (Arber and Cooper 1999; Case and Paxson 2005; Yount and Agree 2005). This has been considered a paradox since women tend to have longer life expectancies (Christensen 2008; Rieker and Bird 2005). The increased likelihood of being a care-giver and the concomitant demands of care-giving may be contributory factors to the poor health and wellbeing associated with older women.

Our findings suggest that care-givers tend to be in good physical health, but may suffer emotionally from the demands of care-giving. This may be a reflection of a selection effect into the study, as those older people who were in good physical health themselves were most likely to be able to carry out the physically demanding task of being a care-giver. Findings from a Kenyan longitudinal grandparent study (Ice *et al.* 2010) appear to suggest that over time care-giving may lead to poorer physical health. Ice *et al.* (2010) demonstrated that while cross-sectionally care-giving older people had better health than non-care-giving older people, over time and adjusting for perceived stress, care-givers experienced declining health. Even though non-care-givers experienced a similar decline in their health status, this was not statistically significant. This could partly be attributed to a confounding effect of non-care-givers being more likely to be receiving care and less likely to have poor emotional wellbeing than care-givers as we show here. Older people provide care to their adult offspring and grandchildren due to, but not limited to, ill-health in young adults, high adult unemployment levels, in response to adult's labour migration and at the death of adult offspring (Ardington, Case and Hosegood 2009; Hosegood, Benzler and Solarsh 2006; Ssengonzi 2007).

The financial strain of care-giving may also be a source of emotional ill-health. As reported elsewhere, care-giving does have financial implications for older people (Ardington *et al.* 2010). Approximately 42 per cent of adults being cared for in our study had been contributing to household income before they became ill, compared to 23 per cent of the children being cared for. As such, care-giving is not only likely to impact upon physical functioning ability but also on the financial situation of older people. Care-givers in our study were more likely than non-care-givers to report their financial situation had deteriorated compared to three years ago, which may be indicative of the financial strain associated with care-giving. This could to a large extent explain why care-givers to adults were significantly more likely to be in poor emotional wellbeing adjusting for age, sex, grant receipt, education, marital status and household living arrangements. An overwhelming majority of study participants belonged to households of which they or another older person were the head. Household headship comes with certain expectations and responsibilities of care and support. This expectation of care and support for the household comes into sharp focus when considering that the majority (91%) of study participants were not working. It therefore was no surprise that most participants cited old-age pension grants as their main source of income. Old-age pension grants in South Africa are an important source of cash income, especially in rural households. Older people without a steady source of income or with limited resources are likely to suffer emotional ill-health. In a study in Johannesburg, South Africa that examined the importance of government cash-transfers in mental health (Plagerson *et al.* 2010), it was found that cash-transfer recipients had a lower risk of common mental disorders.

Using three waves of survey data in the United States of America (USA), Rozario, Morrow-Howell and Hinterlong (2004) showed that older care-givers with multiple roles reported better health than non-care-givers, in support of the role enhancement hypothesis; there was no evidence in support of the role strain hypothesis. In contrast, Reid and Hardy (1999), using data also from the USA, showed that although those with multiple roles may have poorer emotional wellbeing in terms of reported depressive symptoms, after adjusting for demand and satisfaction derived from such roles, having multiple roles had no effect on care-givers' wellbeing. In our study, caring for both adults and children has similar effects on older people's health and wellbeing to caring for either adults or children only; although the odds ratios suggest stronger associations for care-giving to adults than to children. This may indicate that care-giving to adults is generally more taxing than care-giving to children. Further, being a care-giver to adults and to children simultaneously is likely to be associated with higher risks of poorer health than care-giving to children or adults only. However, these older people are not only care-givers; in many cases they contribute vital income to the household wellbeing and, as heads of households, are expected to provide guidance to their household members and to participate in community activities. Hence, our findings suggest a complex mix of role enhancement and role strain associated with care-giving among older people. As noted by others (Chepngeno-Langat *et al.* 2011), the consequences of care-giving on the health and wellbeing of older people depend on various factors which may mediate how care-giving impacts health outcomes.

There are two pathways through which care-giving could lead to ill-health and wellbeing among older people. The first is through a shortage of resources to meet household needs. When older people take on primary care responsibilities in severely HIV-affected communities, it is usually due to HIV-related illness and death and thus there is usually a decline in household income per capita. In South Africa, where many older people are reliant on old-age pension grants to meet the needs of the household, taking on care responsibilities could overstretch the limited pension grant to cater for the needs of adults and children for whom the grant is not intended. The major challenges that care-giving older people face include limited financial support, inability to provide adequate food and clothing, paying for orphaned children's school fees, medical expenses for ailing adults or children and daily physical care (Agyarko, Kalache and Kowal 2000; Nyambedha, Wandibba and Aagaard-Hansen 2003). These challenges could lead to increased physical, financial and emotional stress (Agyarko *et al.* 2002; Schatz 2007) and ultimately are likely to lead to ill-health among older people (Schatz and Gilbert 2012). The second pathway through which care-giving could lead to poor health and wellbeing among older people is through care-giving acting as a psychological stressor. According to the stress process model (Pearlin *et al.* 1990), day-to-day care-giving with its associated challenges may lead to psychological strains in care-givers. It has been shown that physiologically, long periods of stress increase cortisol and catecholamine (Sapolsky 1996, 1999), which are risk factors for cardiovascular diseases and glucose intolerance, among other health problems (McEwen 2001; Sapolsky 1996). Older people are said to be highly susceptible to these effects of chronic stress (Sapolsky *et al.* 1987). We found that care-giving is associated with higher odds of poor quality of life both from our computed quality of life index and from participant's own self-perceived quality of life. Should this care-giving stress among older people be sustained for a long period, they are likely to suffer serious health problems such as hypertension, cardiovascular disease, stroke and even death according to the stress process models.

Our results show that HIV-infected participants were less likely to be receiving filial care than HIV-affected older people and that older people who were in receipt of care were less likely to be in good health on all health measures considered, except for self-reported health status in the last two weeks. The large-scale roll-out of antiretroviral therapy (ART) since 2004 has had tremendous impact on morbidity and mortality of HIV-infected people world-wide (Joint United Nations Programme on HIV/AIDS (UNAIDS) 2011). Although it has generally been assumed that HIV-infected people would have poorer health than their HIV-uninfected counterparts (Justice 2010), a small body of emerging evidence suggests that the former may actually be in better health than the latter. In South Africa, HIV-infected older people had better functional ability (*p*<0.001), quality of life (*p*=0.011) and overall health status (*p*=0.001) than HIV-affected uninfected older people (Nyirenda *et al.* 2012). Similarly, findings from Uganda showed lower disability and a better composite health score among HIV-infected than HIV-uninfected older women (Scholten *et al.* 2011). This finding has been attributed to the enhanced health care and other support devoted to the HIV care programme (Houlihan *et al.* 2011; Nyirenda *et al.* 2012). In a study in Cambodia comparing participants on ART to those on diabetes treatment (both chronic conditions), only those on ART additionally received other social support, food, transport reimbursement to and from the clinic, and free medications (Men *et al.* 2012). Patients on ART in South Africa, as per Department of Health guidelines, are routinely monitored, including home visits if they miss a number of scheduled clinic visits, and any opportunistic infections are promptly treated (Houlihan *et al.* 2011). In addition, initiating ART is a qualifying criterion for disability grant, whose combination with effective treatment contributes to improved health outcomes (Knight, Hosegood and Timæus 2013). Our finding that HIV-infected participants are less likely to be care-recipients than HIV-affected participants could therefore be associated with them generally being in better health. As expected, care-receiving is strongly associated with poor health; it remains to be seen, however, whether over time the emotional wellbeing of care-recipients improves.

### Limitations of the study

This was a cross-sectional study hence we cannot make inferences of causality between care and health status. Furthermore, our results are not generalisable to the overall older people population in South Africa, since we purposefully divided study participants into four strata that could easily be categorised into HIV-infected and HIV-affected groups. Lack of a control group that was neither HIV-infected nor HIV-affected was another limitation of this study. Our findings could, however, be extended to the population of HIV-infected and HIV-affected older people in similar rural settings. We could not disentangle a healthy selection effect from the data, in that participants in our study are likely to be survivors of their respective cohorts. Consequently, some of the association between being a care-giver and good physical health observed in this study may be accounted for by this healthy selection effect. Only in a longitudinal study would we be able to observe whether the health and wellbeing of care-givers declines over time.

All the health measures used here are self-reported. There may have been some reporting desirability bias; older people who are giving care to orphans and HIV-affected adults may overstate their ill-health and wellbeing status owing to the presentation of caring for orphaned children and HIV-affected adults as a burden. The fact that our results show care-giving to be associated with good physical functioning ability but poorer emotional wellbeing gives us confidence in our results; even if the study participants overstated their ill-health, our findings are consistent with the expectation that to be a care-giver one should be in relatively good physical health but may be emotionally stressed by the care-giving role. The primary focus of this study was on older people providing care to adults and children. Care-giving from one older person to another was not explored. Studies from the USA suggest older people caring for their peers tend to have multiple limitations in activities of daily living (Ice *et al.* 2010; Minkler and Fuller-Thomson 1999). Thus the findings presented here may only be a partial reflection of the burden of care-giving among older people and its associated effects on their health and wellbeing.

Other household living-arrangement factors such as household composition, other persons contributing to household income, and the number and orphanhood status of children in the household have not been included in this analysis. This will be the subject of a future analysis, which will explore in detail household living arrangements and their implications on intergenerational flow of care and support using longitudinal surveillance data. In spite of these limitations, this study makes an important contribution to the limited knowledge of the associated effect of care-giving and care-receipt on the health and wellbeing of HIV-infected or -affected older people in rural South Africa.

## Policy implications and recommendations

There is need for increased support to care-giving older people, who we show to report good physical functioning health but poor emotional wellbeing, and who over time are likely to suffer overall ill-health (Ice *et al.* 2010; Wilson *et al.* 1991). As demonstrated by a pilot study in the Eastern Cape province of South Africa, a community-based health education intervention can help older care-givers of HIV-infected grandchildren and adult children to manage their care-giving tasks (Boon *et al.* 2009). There is, however, need for a community-based intervention of routine mental health screening particularly of older care-givers, so that appropriate psycho-social support and health care may be provided to those with poor emotional wellbeing. As suggested by others, such an intervention may be helpful in broadening uptake of existing mental health services in the primary health-care system (Petersen, Baillie and Bhana 2012; Petersen *et al.* 2009).

We also suggest that the government should consider extending the old-age pension programme to all older people aged 50 years and above, rather than the current age threshold of 60 years, since care-giving in many instances starts considerably before age eligibility for the programme. Extending old-age pension grants to all older people, particularly care-givers (to adults or children), may help to reduce the likelihood of being in poor quality of life, as previous research has shown old-age pension grant recipients tend to have a lower risk of mental ill-health (Plagerson *et al.* 2010).

Family, especially an older person's children and grandchildren, are an important source of care and support, and those who are childless or without a circle of immediate family members are likely to be vulnerable when in need of care and support. In a society where institutional care facilities are non-existent and not normative, there is a need to encourage community leaders, religious organisations and neighbours to come to the assistance of such vulnerable older people in activities like drawing of water, cooking and cleaning their homestead. Our findings showed that less than 2 per cent of older people received care and support from church, community volunteers or neighbours. In many traditional African societies older people were revered and young people, whether related or unrelated to the older person, were expected to assist them. Such value systems need to be promoted in contemporary societies which are increasingly becoming individualistic.

Finally, the two pathways of economic stressor or psychological stressor through which care-giving could lead to ill-health and wellbeing are by no means mutually exclusive (Ice *et al.* 2010; Pearlin *et al.* 1990). When designing interventions for care-giving older people, it is therefore important to intervene both at the socio-economic and the emotional wellbeing levels.

## Conclusion

Nearly two-thirds of study participants in this rural setting with high HIV prevalence were care-givers to at least one adult or child; this high care-giving burden is of concern. The severe impact of HIV and adverse economic circumstances among adults, on one hand, and increasing frailty and HIV infection among older people, on the other hand, are likely to lead to a greater number of older people simultaneously providing and needing care. Given the effect of care-giving on the health and wellbeing of older people, it is important that policy makers and practitioners put in place interventions to support older care-givers.
